# Damage detection of road domain waveform guardrail structure based on machine learning multi-module fusion

**DOI:** 10.1371/journal.pone.0299116

**Published:** 2024-03-15

**Authors:** Xiaowei Jin, Mingxing Gao, Danlan Li, Ting Zhao

**Affiliations:** 1 School of Energy and Transportation Engineering, Inner Mongolia Agricultural University, Hohhot, 010018, Inner Mongolia, China; 2 College of Energy and Transportation Engineering, Inner Mongolia Agricultural University, Hohhot, 010018, Inner Mongolia, China; Sichuan University, CHINA

## Abstract

The current highway waveform guardrail recognition technology has encountered problems with low segmentation accuracy and strong noise interference. Therefore, an improved U-net semantic segmentation model is proposed to improve the efficiency of road maintenance detection. The model training is guided by mixed expansion convolution and mixed loss function, while the presence of guardrail shedding is investigated by using partial mean values of gray values in ROI region based on segmentation results, while the first-order detail coefficients of wavelet transform are applied to detect guardrail defects and deformation. It has been determined that the Miou and Dice of the improved model are improved by 8.63% and 17.67%, respectively, over the traditional model, and that the method of detecting defects in the data is more accurate than 85%. As a result of efficient detection of highway waveform guardrail, the detection process is shortened and the effectiveness of the detection is improved later on during road maintenance.

## Introduction

Guardrails are important components of the road infrastructure, primarily along the roadside to maintain a reasonable width of the net area so that vehicles are prevented from straying off the road or into the opposite lane [1]. In addition to protecting the safety of drivers and passengers, both sides of the road edge can also remind drivers to identify the direction of travel in order to improve driving safety. Further, the installation of a reasonable and attractive road guardrail not only improves the aesthetics of the road, but also increases the safety of the roadway and improves the traffic flow. Due to the impact of vehicles and the weather, highway guardrails may deform or break; and steel and aluminum alloy guardrails may also deteriorate over time due to corrosion layer wear and material aging. A long-term aging and shedding process will gradually erode the safety of the guardrail. Therefore, the detection and judgment of highway guardrail diseases is of paramount importance in preventing major traffic accidents and ensuring the safety of public infrastructure.

Deformation, damage, and corrosion of guardrail layers are the most common indicators of infrastructure detection [2]. A traditional manual statistical method remains the standard for highway guardrail inspection in the present day. There are still many limitations regarding the number, length, and quality of guardrails and other aspects of the work in terms of statistics. Furthermore, manual inspections require long periods of time, are inefficient, and will have an error rate which can be improved by reducing manual fatigue. As a means of overcoming these limitations, artificial intelligence and computer vision technologies are applied to road maintenance detection in order to obtain the number, length, and quality of highway guardrail information, while detecting potential defects in the guardrail, thus providing reference material for maintenance personnel, as well as protecting driver safety. Currently, highway waveform guardrail research is characterized by two major components, on the one hand through the use of LIDAR and scanning lines for extracting and detecting road waveform guardrails, and on the other hand through the use of machine vision for obtaining the characteristics of the detection method.

Using corner point features and height features, HaoZhu segmented point clouds into straight lines for road guardrail extraction using LiDAR and scan lines [3,10]; Using multilevel filters and improved spatial clustering, Jianlan Gao et al. enhanced the accuracy of guardrail structures by screening them with a great deal of accuracy [4]. Ming Huang studied the highway sites using laser scanning as a result of laser scanning. According to Ming Huang, binary coded voxels were used to study the highway sites, and clustered slicing of the highway guardrail was used to identify it based on its features. This resulted in highly accurate and efficient identification of the highway guardrail [5]. However, the effect of slicing is influenced by the sample environment.; Yuanwen Yue et al. extracted the scanned vehicle trajectory, constructed buffers to remove points far from the roadside, and applied multiple geometric filters and statistical outliers to remove points near the roadside in order to identify guardrail points and calculate their perpendicularity to enhance the detection. The algorithm is very accurate at detecting and mapping guardrails, however, the process for doing so is very complex, time consuming and inefficient [6]; A. Broggi uses radar and vision data to identify uninterrupted diagonal lines in the radar sensor’s region of interest to increase the speed at which the vehicle detection algorithm can be carried out. This method has significant advantages in terms of time and detection efficiency, but also has substantial limitations [7].

In the field of machine vision, methods of shallow machine learning and deep machine learning are primarily used for detection. Shallow machine learning methods rely primarily on digital image processing. Alexander Seibert obtained three-dimensional information about guardrails using a forward grayscale camera and Lucas and Kanada trackers, but the accuracy of guardrail detection was low [8]; In Hao Zhu et al., clustering was employed to detect guardrails by extracting feature points, filtering unnecessary guardrail points, and adaptively extracting regions of interest (AROI) using a Kalman filter algorithm. However, there are many interference factors involved in filtering unnecessary feature points from this method, resulting in poor extraction accuracy [9,10]; Hao Zhu has also proposed a method combining HOG and LBP multi-feature fusion after PCA dimensionality reduction to detect guardrails, and the accuracy is enhanced significantly, but the speed is not guaranteed [11,12]; Zihao Xu describes a new Lazy Snapping algorithm based on the Graph Cut idea to segment guardrails in complex environments, but the algorithm needs to rely on too much human-computer interaction and cannot achieve real-time detection [13].

Deep learning and neural network models are the primary methods of deep machine learning. As part of the image preprocessing algorithm, Hao Jin introduced Mask RCNN in the image preprocessing algorithm, which combined Rencent101 as the backbone network with a pyramid network structure for feature extraction in order to achieve guardrail segmentation and detection, but it requires a large number of samples to train [14–16]. He Pu Zheng performed instance segmentation to detect the highway waveform beam guardrail, which resulted in a slow training speed due to the amount of training data and the complexity of the model [17]. According to Li Zhenyu et al., a guardrail recognition system was developed, coupled with improved edge extraction algorithms and linear fitting algorithms, and a binocular vision method was proposed for the automatic detection of guardrail deformation, and it was able to detect guardrail deformations with a detection accuracy of 92.8%, however, their recognition model has a relatively small sample size and the recognition accuracy still needs much improvement [18].

It is anticipated that LIDAR will incur higher costs, digital image processing can no longer be guaranteed as efficient and real-time, and deep learning requires a large number of samples to support the network model. In order to increase the accuracy and speed of highway waveform guardrail extraction under the premise that the data set is limited, the U-net network is employed as a model to extract highway guardrails. There is a high degree of effectiveness in segmenting small targets that can be attributed to the U-net model proposed in 2015. As a result of its pixel-level calibration, the U-net model has a simple network structure that can be trained on a small number of samples. The trained samples also have higher accuracy due to the simple network structure of the U-net model.

A U-net network model is commonly used within the medical field at present, however it has been gradually introduced into other segments of segmentation fields. Zhu Suya detects bridge cracks by U-net shallow network, and the experiment shows that the width of the detection is measured accurately and can meet the needs of the application [19]; Song Tingqiang extracts road targets from remote sensing images using an improved U-Net network. The results indicate that the improved network model has high segmentation accuracy and is applicable to multiple applications [20]. A combined residual network model and U-net network model was developed by Zhengxin Zhang to extract roads from aerial impacts. This novel network model performed more effectively than other deep learning-based models for road extraction [21]; According to Wang et al., the DDU-Net model was developed in which the U-net network was enhanced to extract roads from remote sensing images by adding dilation convolutional attention and block attention modules, and the results showed that the proposed model improved both the MIOU and F1 scores[22]; Wei Fang identified road cracks by Deeplab V3+ model in semantic segmentation, and the average intersection ratio of the obtained segmented images could reach 81.31% [23]; Yuchun Fang The proposed method outperforms previous restoration methods by mixing dilated convolution and spectral normalization to focus on face restoration tasks [24]; Chuang Yu obtained the shape feature size of fish in real scenes by combining an improved U-net network model with a least-squares linear fitting method, and the results showed that the relative error of the improved method was 0.37% reduction [25]; Zhouzhou Zheng used an improved U-net network model to detect cracks on the surface of jujube. The results revealed that the segmentation method yielded higher accuracy and detection efficiency than the U-net network model [26]. Several aspects of U-net network application were successfully implemented with good results and accuracy, suggesting that U-net network has been applied in many fields and presents a great opportunity for this study to utilize U-net.

Existing guardrail segmentation methods and datasets are limited, and all existing guardrail segmentation models are trained using in-vehicle datasets. Furthermore, all existing detection methods require a substantial amount of data for training. Consequently, this study proposes an improved U-net-based segmentation model for the segmentation of guardrails. The hybrid loss function is used to guide the model to learn different data types, and the HDC module is added to learn the features of different sensory wild targets, which is trained on the self-constructed dataset of UAV aerial photography and part of the public dataset with the presence of guardrail targets, and based on the segmentation of the results obtained, guardrail defects are detected and determined. The experimental results show that the method proposed in this study is effective in performing both guardrail disease segmentation and detection. The proposed method solves the problem of insufficient existing datasets and the need for large volume data training by adapting to both in-vehicle and UAV collected image data with a small sample dataset.

This paper proposes an effective method for detecting and segmenting highway guardrails, which can aid in road inspection and disease maintenance. The contributions of this paper are outlined below:

1. Firstly, the U-net network structure has been improved with the HDC module to better suit the shape and data characteristics of highway guardrails and enhance its ability to segment them, while also considering the characteristic analysis of highway guardrails.
2. secondly, datasets based on UAVs and vehicle-mounted road barriers have been created as a means of adapting to different types of acquisition tools.
3. In addition, datasets based on UAVs and vehicle-mounted road barriers have been created as a means of adapting to different types of acquisition tools.

## Proposed framework

It is important to detect road waveform guardrail defects automatically to prevent further damage to the guardrail, but it is also a crucial part of the daily inspection of the road. In this study, we used semantic segmenting in the U-net neural network for highway waveform guardrail ROI region extraction, and according to the characteristics of different types of defects, the detection method is proposed.

First, the UAV was used to collect images from various angles within actual scenes to produce a dataset for ROI region extraction. Secondly, the U-net network was combined with hybrid expansion convolution and hybrid loss functions to enhance segmentation accuracy of large field of view target images to generate ROI binarized images with high precision.

**Fig 1:**
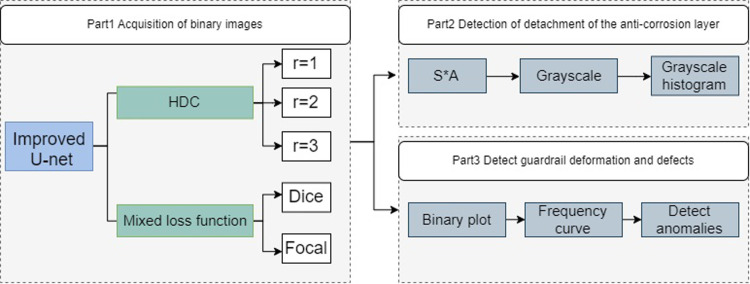
Overall flow chart, the process structure.

We propose two types of defect determination methods for detecting corrosion layer peeling and guardrail structure deformation. Defect one is determined by calculating the grayscale histogram of the segmented ROI area, and judging whether there is anticorrosive layer shedding on its surface according to the range and size of its grayscale value; defect two is detected by first correcting the binarization and performing random transform in the direction perpendicular to the Hough line to get the frequency histogram in that direction, and finally performing first-order wavelet transform by discrete wavelet transform to get the high frequency band and low-frequency band, and determine whether there is a defect location by the size of the detail coefficient. As part of the highway waveform beam guardrail system, defect detection and prevention is possible, information about guardrail defects can be efficiently accessed, road maintenance personnel will be more efficient, road safety will be improved, and the driver’s safety will also be greatly improved (Fig 1).

## Materials and methods

### Improved U-net network model

**Fig 2:**
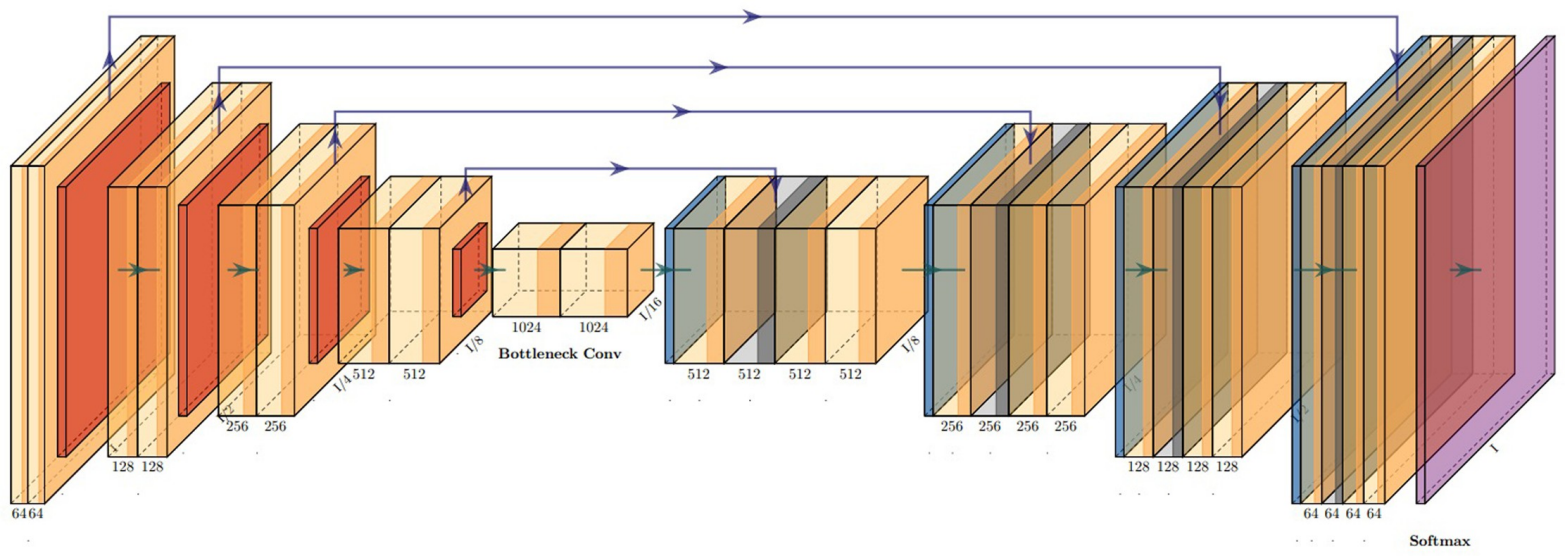
Traditional U-net network structure.

As highway guardrail backgrounds are complex and similar, traditional digital image processing techniques have certain disadvantages. Deep learning models such as CNNs require a large number of samples for training. Whereas the U-net network model is a pixel-level fully convolutional neural network with no need for large numbers of samples to be trained. It can be trained by using small samples and can be convolved on data of any size. The U-net model is a pixel-level fully convolutional neural network, which can be trained with small sample sets (Fig 2).

**Fig 3:**
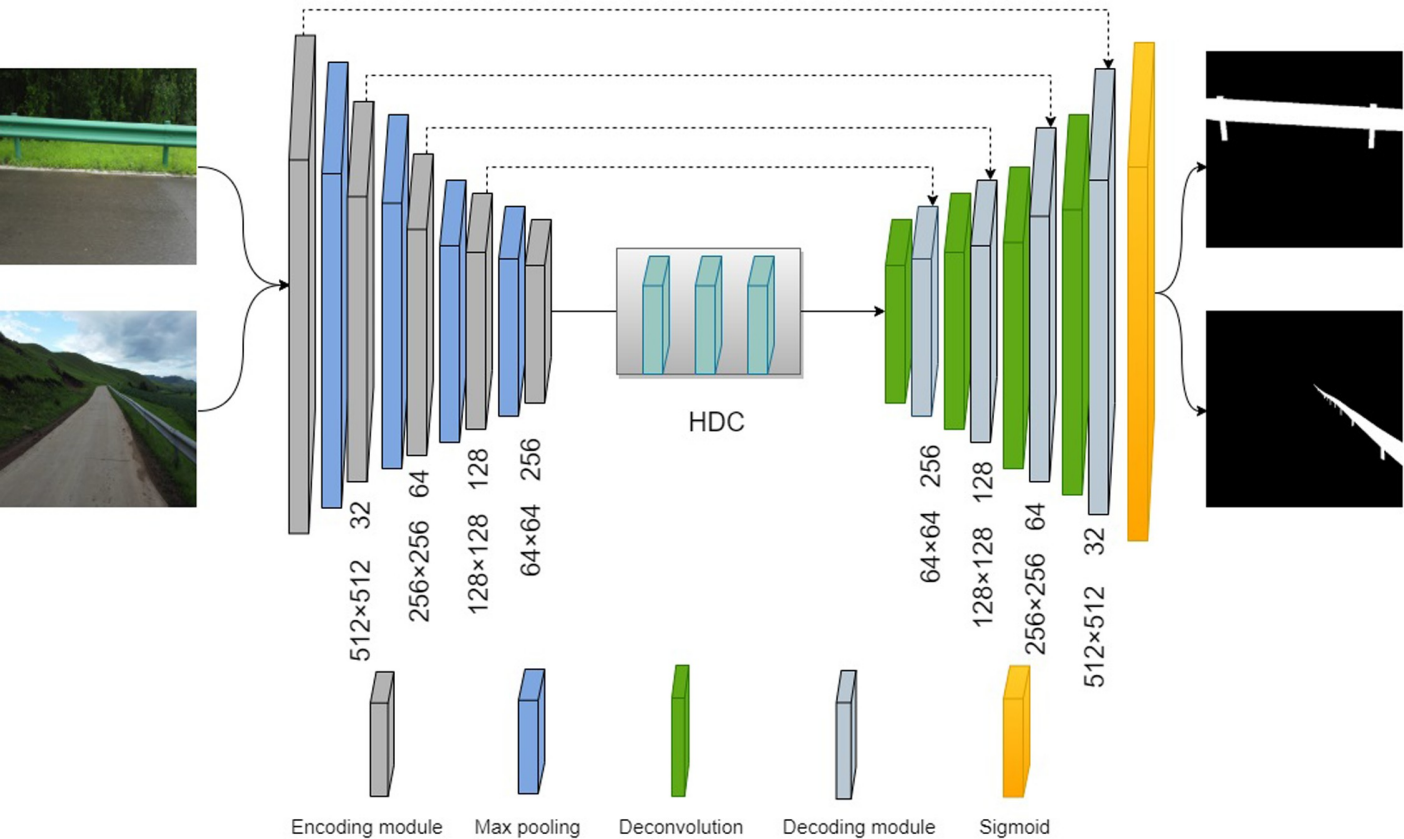
Improved U-net network structure diagram.

The main components of this path are the symmetric shrinkage and expansion paths. With the down-sampling of the shrinkage path, we can increase the feature map and capture more contextual information, while the expansion path improves the accuracy of segmenting the target and compensates for boundary loss. An improved U-net neural network is proposed to capture more contextual information with accurate localization and improve the model’s ability to extract image features. Firstly, to ensure the model trains correctly during the training process, a loss function combining dice coefficients and focal loss is used. Second, instead of conventional convolution in the original model, a hybrid expanded convolution is utilized in the bottleneck module. It is possible to increase the perceptual field while maintaining the feature space resolution, thereby improving the model’s ability to extract features from images. U-net’s structure after the improvements is shown in (Fig 3).

#### Hybrid loss function

The segmentation of small targets has proven to be a challenging task in semantic segmentation, and the unbalanced sample categories in the training dataset are one of the main reasons for reducing model training accuracy. It is possible to apply data enhancement techniques to make the training set more balanced by changing the samples. However, as the dataset lacks diversity, it does not contribute significantly to improving the model. Therefore, the number of target images for the different scene categories is adjusted to ensure the accuracy of the model training. Table 1 below illustrates the ratio of target images under different scene categories.

**Table 1:** Proportion of different scene categories in the data.

Label	Label 0	Label 1	Label 2	Label 3
Percentage/%	23%	40%	20%	17%

Different shooting angles in the dataset result in ROI regions with different volume ratios. To reduce the impact of sparse classification errors on model training and to enhance the penalty associated with sparse classification errors, a hybrid loss function is used to bootstrap the model. Dice loss is mainly used to alleviate the problem of unbalanced distribution of positive and negative samples of voxels, and is used to measure the similarity between two samples. A smaller value indicates a greater similarity between two samples. This measure evaluates the similarity between two samples. By removing pixels from the predicted image that aren’t activated by the labeled image, this function penalizes the prediction with lower confidence and lowers the Dice coefficient.


$$L_{\text{D}ice}=K-\sum_{k=0}^{K-1}\frac{2P_{TP}(k)}{2P_{TP}(k)+P_{FN}(k)+P_{FP}(k)}$$ (1)

In this case, represents the probability of k being true positive, false negative, or false positive, respectively.

The focal loss function enables the model to focus more on learning poorly classified samples by decreasing the weight of easily distinguishable negative class samples. It also increases the weight of sparse samples.


$$L_{Focal}=\frac{1}{X}\sum_{y=0,1,2,4}\sum_{x=1}^{X}{\left[1-p_{x}(y)\right]}^{\gamma }t_{x}(y)\log\left[p_{x}(y)\right]$$ (2)

As the value of γ directly affects the loss function, the greater the accuracy of the model prediction when γ is determined, but the lower the contribution to the loss function after decay; and the larger γ is, the more obvious this trend of change. Therefore, the focal loss function plays a significant role in solving the sample imbalance problem. In order to solve the problem of category imbalance in the sample, the loss function combining dice loss and focal loss is employed as follows:

$$L_{loss}=L_{Dice}+\lambda L_{Focal}$$ (3)

Based on the results of the experiments and the literature referenced [27], we can conclude that modeling is most effective when λ = 0.5.

#### HDC module

Expanded convolution enlarges the size of a normal convolution to obtain a larger perceptual field using the same convolution kernel unit. Basically, it involves enlarging the convolution kernel without increasing the computational effort or reducing the resolution of the feature map in order to increase the perceptual field. In 2D images, expanded convolution is defined as follows:

$$Y[i]=\sum_{k=1}^{K}u[i+d\cdot k]f[k]$$ (4)

It can be seen that *Y*[*i*],*u*[*i*] is the input information and *f*[*k*] is the output information, respectively, with *f*[*k*] representing the filter with a convolution kernel of *k*, and *d* representing the expansion rate.

The use of dilation convolution in semantic segmentation systems replaces the maximum pooling operation or the stepwise convolution layer operation in order to increase the perceptual field while maintaining the image’s resolution in the feature space. This method is commonly applied to feature maps that have already been downsampled. As a result of the introduction of zeros in the convolution kernel in dilation convolution, fewer pixels are actually involved in the convolution. This results in a tessellation view of pixels in the top dilation convolution layer, where most of the information is lost and there is a phenomenon known as "latticework" [28].

**Fig 4:**
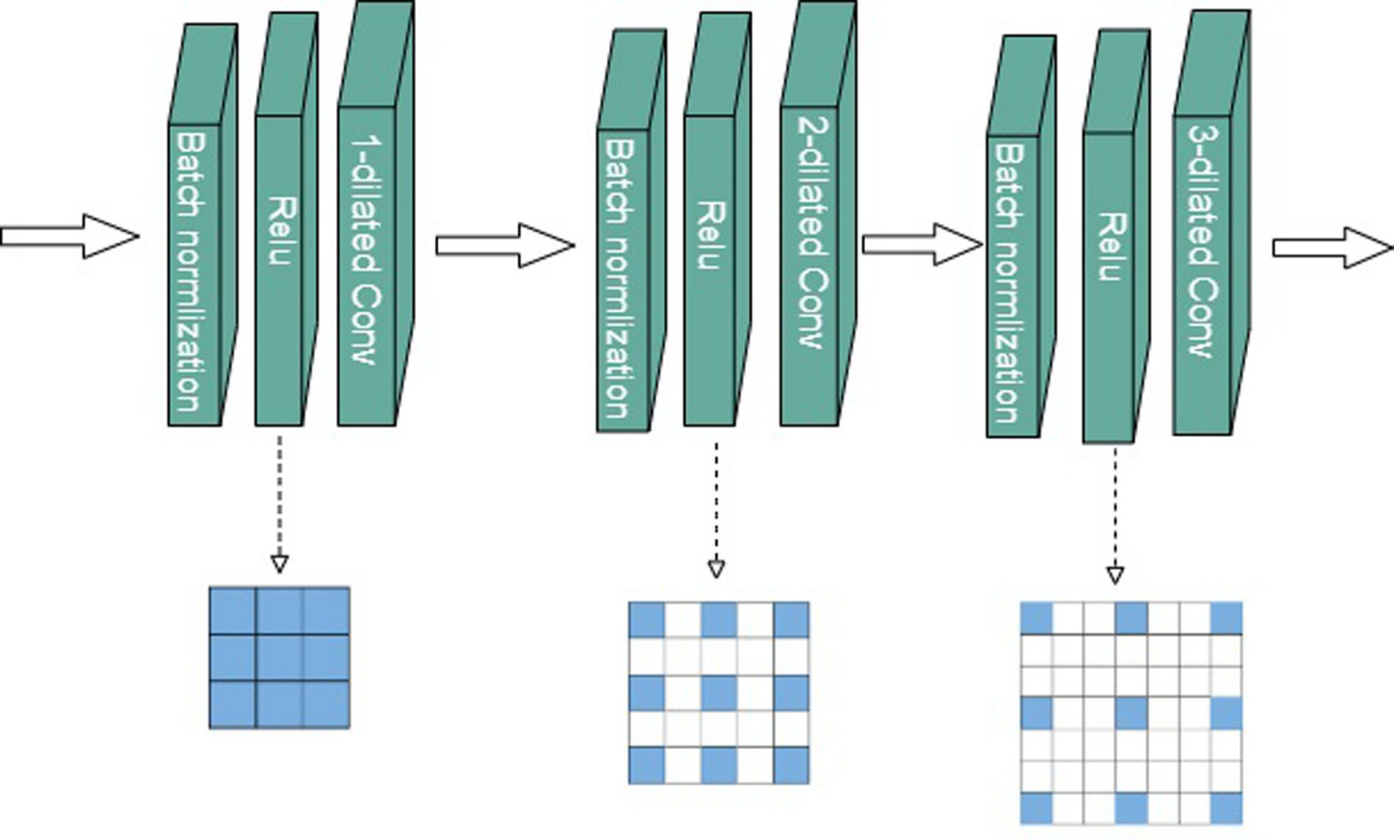
Hybrid expansion convolution structure diagram.

As an alternative to the conventional convolutional network, hybrid expansion convolution is used to enhance the segmentation accuracy of the close-up image, as well as to attenuate the tessellation effect caused by expansion convolution. Three cascaded expansion convolution layers are used as a group, with their rates set to 1, 2, and 3 [28]. An example of a stepped hybrid expansion convolution structure is depicted. This structure allows the top layer to access a greater range of pixel information (Fig 4).

The expansion coefficient of the hybrid expansion *M*_1_ = 1, *M*_2_ = 2, *M*_3_ = 3, the size of the convolution kernel of the model is 3 × 3, which needs to satisfy. The hybrid expansion coefficient needs to satisfy the following equation [29]:

$$M_{i}=Max\left[d_{i},M_{i+1}-2d_{i},M_{i+1}-2\left(M_{i+1}-d_{i}\right)\right]$$ (5)

Since ,the step coefficients used are satisfying the conditions.

**Fig 5:**
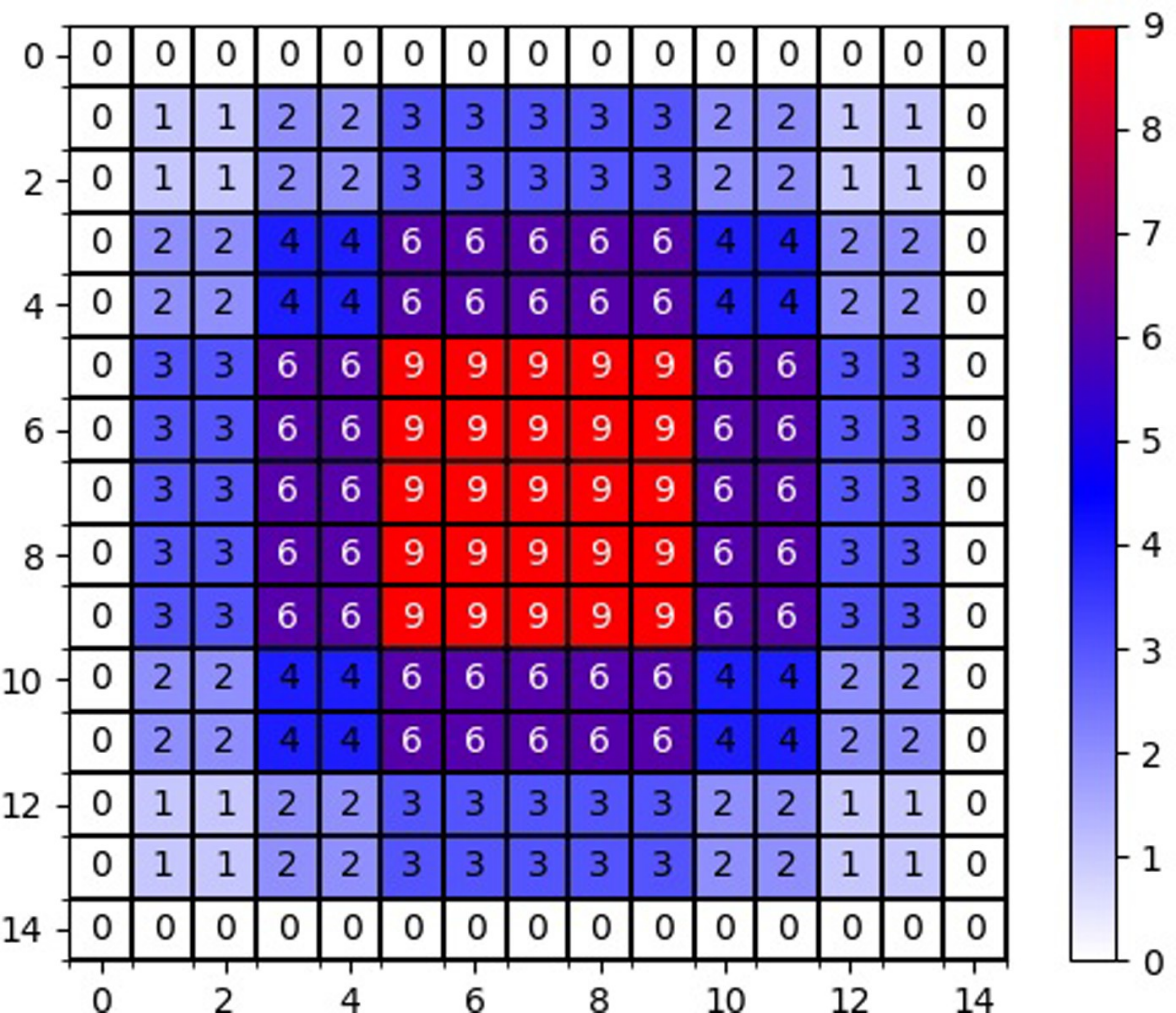
Sensory field changes before and after.

It is possible to recognize images with larger foregrounds using this stepwise hybrid inflated convolution structure. It illustrates the change before and after inflated convolution(Fig 5):

### Model training and analysis

#### Data acquisition

The team collected and labeled the images used in this study due to the absence of official and open datasets of guardrail images in the engineering field. As highway guardrails are usually set up on motorways and their entry/exit roads, plus some low-grade roads on both sides of the road, all the images were taken in the appropriate environment and from different angles. In order to fit better with the actual scenes in practice and to better meet the needs of real-world detection, the road in the former banner of the right wing of Horqin in Xing’an League of Inner Mongolia Autonomous Region was used as the collection section, and the photos were extracted at 20-fps from the captured videos with a resolution of 3840× 2160, including distant and close up views and pictures from different angles. The pictures were adjusted uniformly to 512x512 pixels and were saved as PNG files. Furthermore, the KITTI dataset includes in-vehicle images featuring guardrail target images, which have been selected to enrich the dataset. These images, captured from both vehicle-mounted and UAV perspectives, offer a range of shooting angles and heights that are better suited to real-world detection needs.

#### Data pre-processing

**Fig 6:**
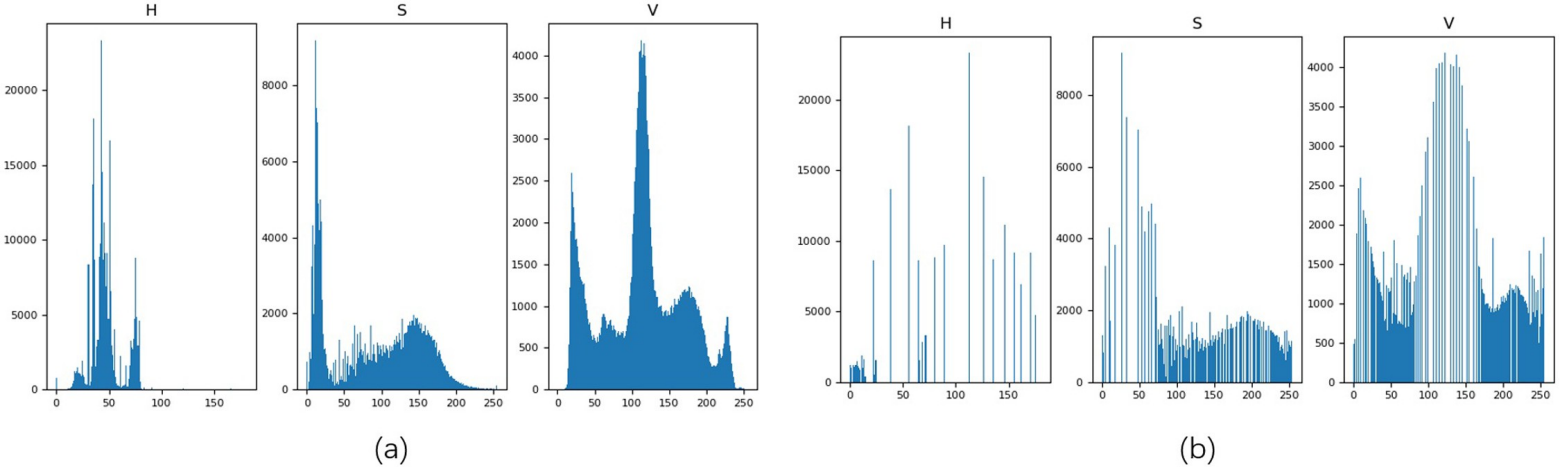
Histogram changes before and after equalization.

Due to the concentration of chromaticity and saturation in the acquired images, which are influenced by weather and light, it is necessary to avoid false detection due to over-saturation of brightness and color. Images are equalized in color space to balance light and illumination effects on the obtained images (Fig 6).

#### Data enhancement

Labelme created a dataset from collected images. Due to a limited amount of data, the dataset was expanded by the method of data enhancement for the collected data in order to achieve maximum balance and diversity in the dataset, and the main enhancement methods and parameters are described below:

1. With probability 0.8, the image will be rotated with a maximum left rotation angle of 10 and a maximum right rotation angle of 10.
2. The left-right swapping of an image is executed with 50% probability.
3. With an 80% probability, the image is enlarged and reduced by 0.85 times its original size

The data was enhanced and supplemented using the methods mentioned above. This resulted in 4411 images, which were trained using 2411 photo sets of graph images with varying angles and backgrounds. The images were then tested using 2000 UAV images obtained from shooting and in-vehicle images from publicly available datasets.

#### Experimental environment

An 11th generation Intel Core i5 processor with 2.4GHz and 16GB RAM was used forutilized as the software environment. Pytorch is used as the framework for conducting the improvement, with Adam as the optimization method. Since U-net is considered a training model with small samples, the learning rate used is 0.01, bitch-size = 4, epoch = 40.

#### Experimental indicators

A quantitative analysis of the performance and accuracy of the algorithm of this paper is conducted using Miou, Dice ,HD (Hausdorff distance) as the basis for the evaluation, and its relevant indexes are calculated as follows:

$$Miou=\frac{TP}{TP+FP+FN}$$ (6)

$$Dice=\frac{2TP}{2TP+FP+FN}$$ (7)

*HD*(*A*,*B*) = max(*h*(*A*, *B*), *h*(*B*,*A*)), Among them

$$h(A,B)=\underset{a\in A}{\max}\left\{\underset{b\in B}{\min}∥a-b∥\right\},h(A,B)=\underset{b\in B}{\max}\left\{\underset{a\in A}{\min}∥b-a∥\right\}$$ (8)

A, B denote the real image and the segmented image, respectively, in the above equations. TN,TP,FN,FP signify true negative, true positive, false negative, and false positive, respectively.

In addition, the F1-score indicator has been added. This indicator provides a comprehensive evaluation of two contradictory measures by averaging the precision and recall rates. It is a composite indicator that reflects both precision and recall rates simultaneously.


F1=2×Precision×RecallPrecision+Recall (9)

As there are currently limited methods for detecting road waveform guardrails, this paper aims to objectively evaluate the proposed model by comparing and analyzing it with U-net, FCN8s, Resnet34-unet, Attention_Unet, and Sobel algorithms.

#### Experimental results

**Fig 7:**
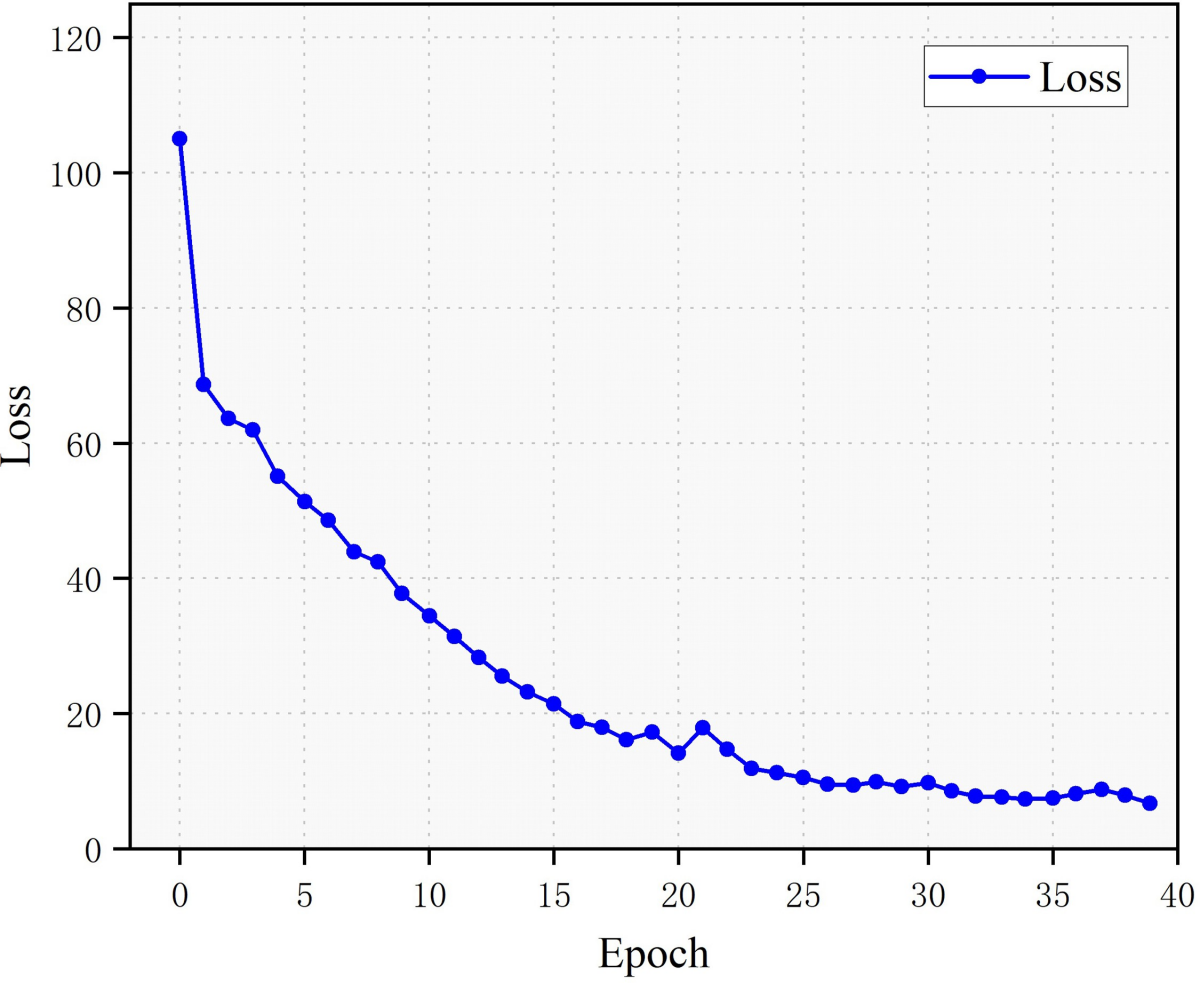
Improved loss, IOU and Dice of model training.

The loss, IOU and Dice after 30 training sessions has improved as a result of the improvement in the loss function (Fig 7).

**Fig 8:**
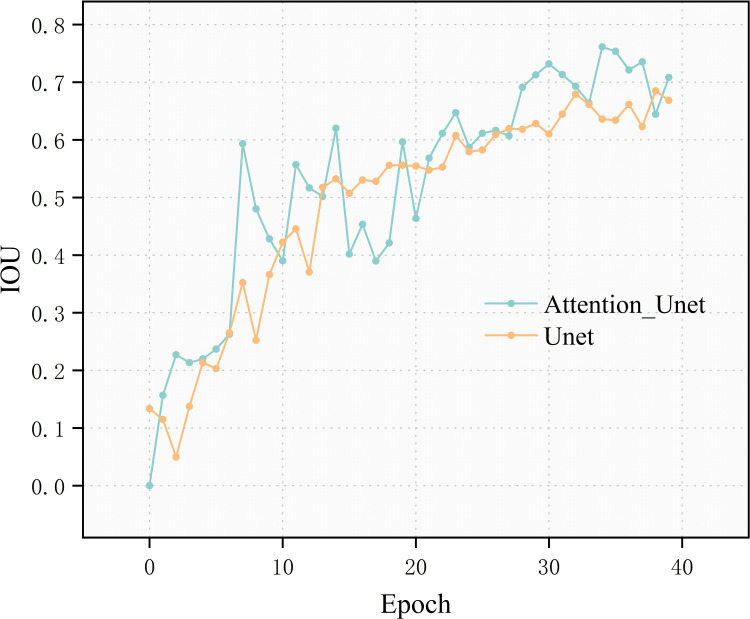
IOU changes across different models.

On the dataset established in this paper, comparison experiments were conducted to verify the effectiveness of the method in segmenting the dataset. The network models compared were U-net, FCN8s, Sobel, Resnet34-Unet and Attention_Unet. The metrics obtained as a result of these tests were compared quantitatively (Fig 8), as shown in Table 2 below:

**Table 2:** Comparison of the metrics between the network model proposed in this paper and other network models.

Index Method	Miou(%)	Aver_Dice(%)	Aver_hd	F1-score
U-net	73.48	74.8	7.12	0.74
Fcn8s	64.44	72.4	7.9	0.76
Sobel	70.8	71.2	8.43	0.70
Resnet34-unet	55.15	66.89	9.03	0.64
Attention_Unet	76.39	75.19	6.91	0.85
Ours	82.11	87.26	5.11	0.90

After comparing the metrics of the aforementioned models, it is evident that the proposed model, Miou, outperforms the others. Specifically, it shows an 8.63% improvement over the traditional U-net and a 17.67% improvement over the accuracy of the Fcn8s model. The Dice coefficient performance is the highest, 12.8% higher than the traditional U-net. This indicates that the predicted results have the highest similarity with the labels. Additionally, the hd of this model is significantly reduced, 2.01 lower than that of the traditional U-net model. This suggests that the algorithm proposed in this paper will be closer to the labelled images in terms of edge segmentation and has better robustness; The F1-score performance is even 0.9, which is 0.16 higher than the traditional U-net. This indicates that the model has higher precision and recall, resulting in better overall performance.

**Fig 9:**
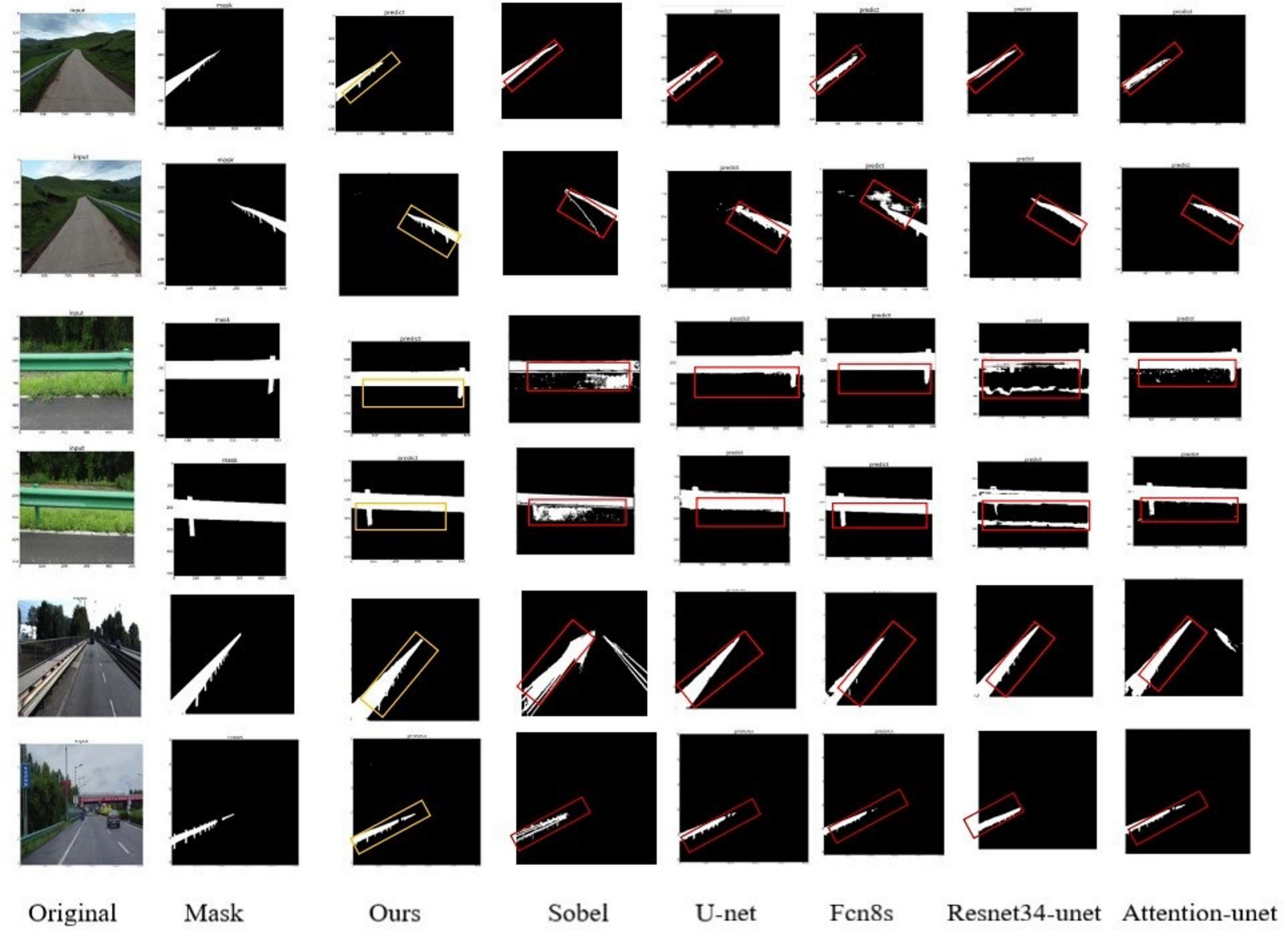
Comparison of segmentation results of each algorith.

For qualitative comparison, we selected images from the test, including UAV images, in-vehicle images, near-view images, and far-view images (Fig 9).

It compares image data from two different scene categories (distant and close-up) and two different acquisition viewpoints (vehicle-mounted and UAV images). The algorithms in this paper are marked with yellow boxes and the other comparison images are marked with red boxes. It can be clearly seen that in the performance of the same image, the algorithm proposed in this paper has the best performance in details and overall, especially in the segmentation of the columns of the guardrail, the performance of other methods’ in the segmentation of the columns is very coarse, and the Sobel algorithm and the Resnet34-unet have omission of checking the lower structure of the guardrail, and even can not guarantee the integrity of the structure. Secondly, Sobel is the worst-performing method, and there is even a lot of connectivity noise, which may have a great relationship with the existence of green vegetation in the background, causing connectivity, and the traditional U-net is inaccurate in detail segmentation, which is shown in the connection between the substructure and the superstructure, but the segmentation results of FCN8s in the near-field image are better, and Mask also basically match, but its performance in the far-field images is not superior. Attention_unet is less effective in long-range image segmentation and the structure is not clear, while segmentation in close-up images will have a lot of noise, mainly concentrated in the substructure of the guardrail. The paper’s model excels in validating in-vehicle images, providing superior segmentation and accuracy compared to other models. As a result, it offers detailed and comprehensive segmentation for both near and far view images of in-vehicle and UAV data.

Further, the times of the above methods were compared and tested, and the results are shown in Table 3.

**Table 3:** Time comparison of several network models.

Method	Time(s)
U-net	6.71
Fcn8s	6.81
Sobel	6.85
Resnet34-unet	13.76
Attention_Unet	14.85
Ours	6.89

Based on the comparison of time, the improved model is only 0.18s slower than the traditional model, and the time difference compared to Fcn8s and Sobel is not significant. According to the above segmentation results, Sobel has no advantage in terms of time or segmentation accuracy. FCN8s is about the same in terms of time as the improved system, although there is still a great deal of work necessary to segment the long-range view. The Resnet34-unet segmentation accuracy and time are not dominant, although the accuracy of the Attention_Unet can reach more than 80%, but the time consumption is also the largest. It has been found that the algorithm proposed in this paper is more accurate and produces better segmentation effects. The addition of the HDC module has not adversely affected the algorithm speed. Therefore, this paper trains the data set on the basis of this model in order to obtain segmented images that will be used to detect defects in the future.

### Guardrail testing

As an important safety appurtenance of the road, the waveform guardrail plays an important role in ensuring the safety of the driver. In accordance with highway maintenance technical specifications [30], highway waveform guardrail maintenance standards are as Table 4 below:

**Table 4:** Comparison of maximum detail coefficient under different defects.

Number	Standard
1	Keep the corrugated beam steel guardrail structure reasonable, safe and reliable.
2	Guardrail panels, columns, column caps, anti-barrier blocks (brackets), solid parts and other components should be complete and free of defects.
3	The quality of the guardrail meets the requirements of the relevant standards.
4	The anti-corrosion layer of the guardrail should not fall off obviously, and the guardrail should be free of rust.
5	The installation line of the guardrail is smooth, without obvious deformation, twist and tilt.

In accordance with the above maintenance standards, (1) and (3) are primarily used in the installation of highway guardrails, whereas the remaining maintenance standards (2), (4), and (5) are primarily concerned with structural defects, deformation and corrosion layer off, and corrosion of the guardrails. Consequently, the detection of highway waveform guardrail defects is primarily concerned with detecting deformation and corrosion layer off two aspects of the guardrail.

#### Detection of highway guardrail corrosion layer off

To detect highway waveform guardrail, the binarized image of the target image must be obtained, and the obtained binarized image must be multiplied with the original image to obtain the RGB image of the ROI:

$$S=C.^{*}A$$ (10)

**Fig 10:**
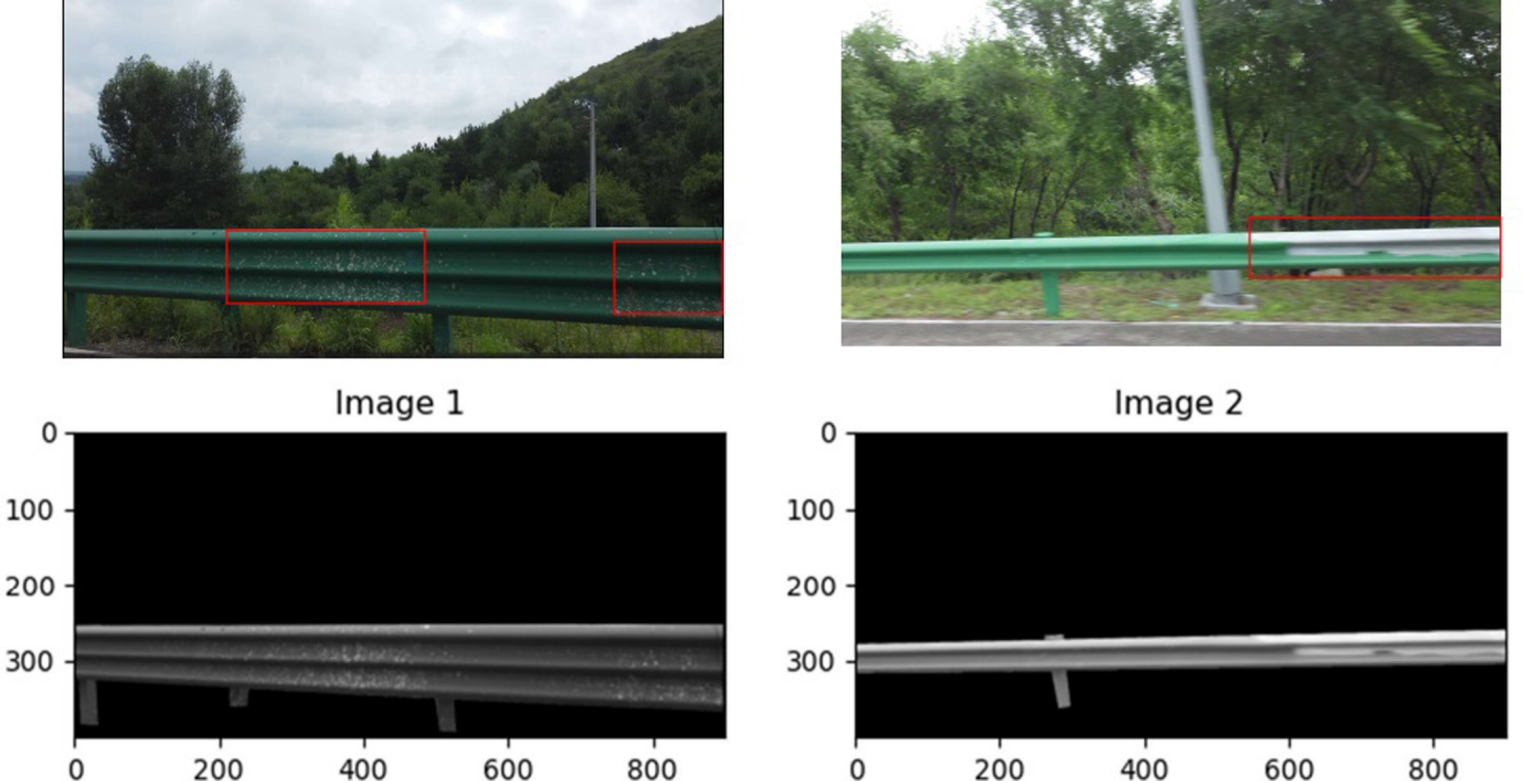
Grayscale map of ROI area.

The three-channel ROI image of C is grayed out to achieve a single-channel grayscale image. C denotes the binarized image, A denotes the original image, and the ROI image resulting from this is shown below (Fig 10):

Based on the grayscale map of the face, the grayscale image has a grayscale value between 0–255, and is easily distinguishable by the naked eye. There are significant differences between the grayscale values of the corrosion layer peeling off and those of the normal part. The grayscale values of the peeling area should be greater than those of the normal part. As the middle grayscale value, 150 is chosen, and the average of all pixels with grayscale values greater than 150 is calculated as the partial average coefficient. Additionally, the partial average coefficients of the normal guardrail and the off guardrail are calculated.

**Fig 11:**
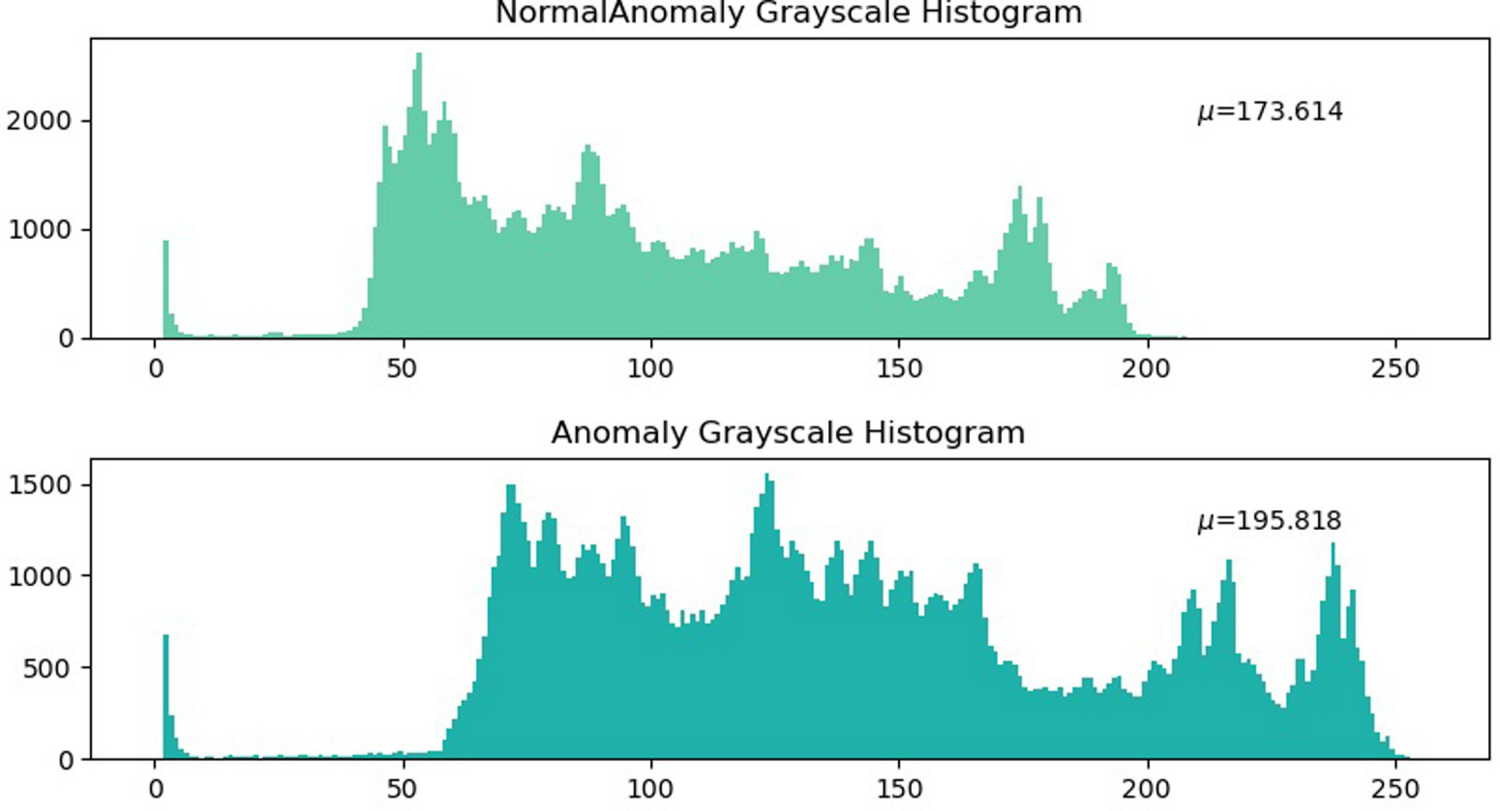
Comparison of the histograms of Image 2.

Thus, the grayscale histograms of the normal guardrail image and the guardrail image with shedding were plotted separately along with corresponding partial average coefficients (Fig 11).

**Fig 12:**
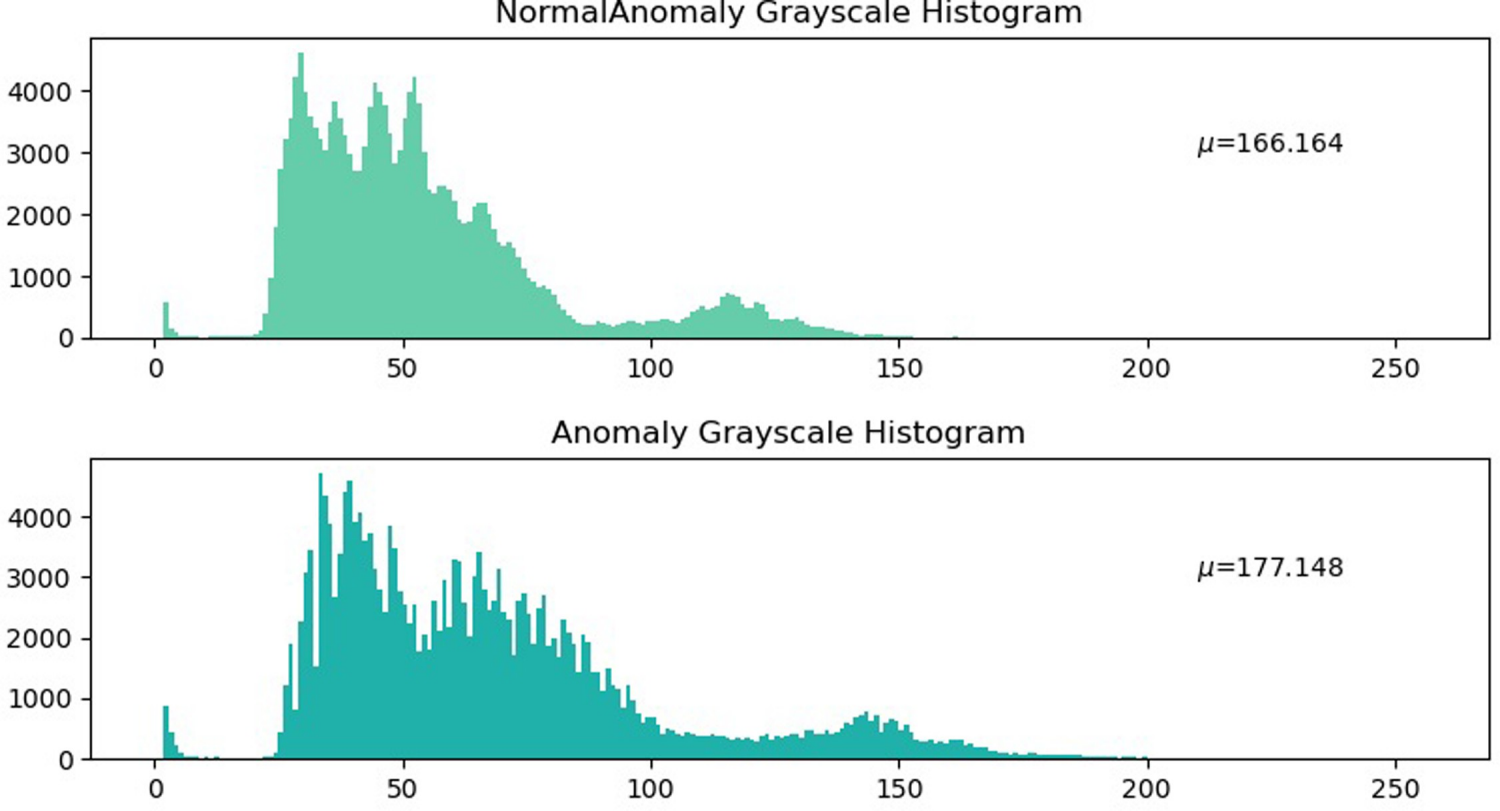
Comparison of the histograms of Image 1.

Based on the grayscale histogram above for Image2, the image with peeling exhibits a higher average bias average coefficient because more pixels are exposed in the brightness area when the corrosion layer peels off, resulting in a larger bias average coefficient for the image with peeling (Fig 12).

For Image1, the problem is the speckle type corrosion layer of off, as can be seen from the gray map, off the location will appear bright, which is also reflected in the gray histogram. There is an image of anti-corrosion layer shedding at 150 pixels, and the partial mean of 177.148 is also higher than the normal μ = 166.164. Due to this, a partial mean value can be used to establish whether there are defects on the surface of a highway waveform beam guardrail, such as corrosion layers that are shed and rust that is present.

#### Detection of highway waveform guardrail missing and deformation

**Fig 13:**
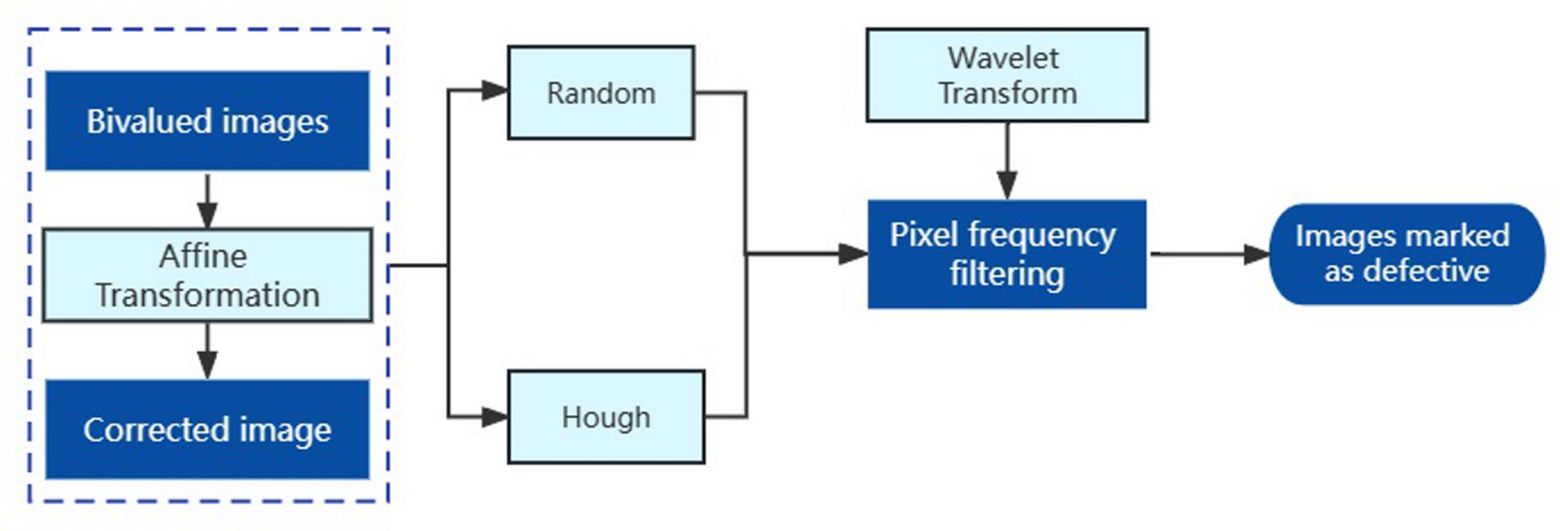
Flow of defect detection.

In order to correct the pixel interference caused by different angles, the binarized image of the target image is subjected to affine transformation. To obtain contour pixel frequency filtering of the image, the corrected image is projected using Hough straight line detection and Random transform. The wavelet transform is used to detect the interval in which the filtering is abnormal to obtain an image of the guardrail marked with defects (Fig 13).

#### Get frequency filtering

On the basis of the ROI binarized image obtained from segmentation, the centroids of the largest contours as well as the width (W) and height (H) of the target region are calculated, as well as their coordinates after correction:

[0,0],[W+1,0],[W+1,H+1],[0,H+1]

Based on the coordinates of the selected image, the affine transformation matrix is calculated as follows.


$$M=\left[\begin{array}{ccc} \frac{9.647}{e} & \frac{-3.365}{e} & 7.85e\\ \frac{3.73}{e} & \frac{9.735}{e} & -1.86e^{2}\\ \frac{3.57}{e^{5}} & \frac{4.02}{e^{5}} & e \end{array}\right]$$

As a result, the affine transformation of a pair of corresponding coordinates is performed according to the following relations:

$$\left[\begin{array}{l} x^{\prime }\\ y^{\prime } \end{array}\right]=M\left[\begin{array}{l} x\\ y\\ 1 \end{array}\right]$$

**Fig 14:**
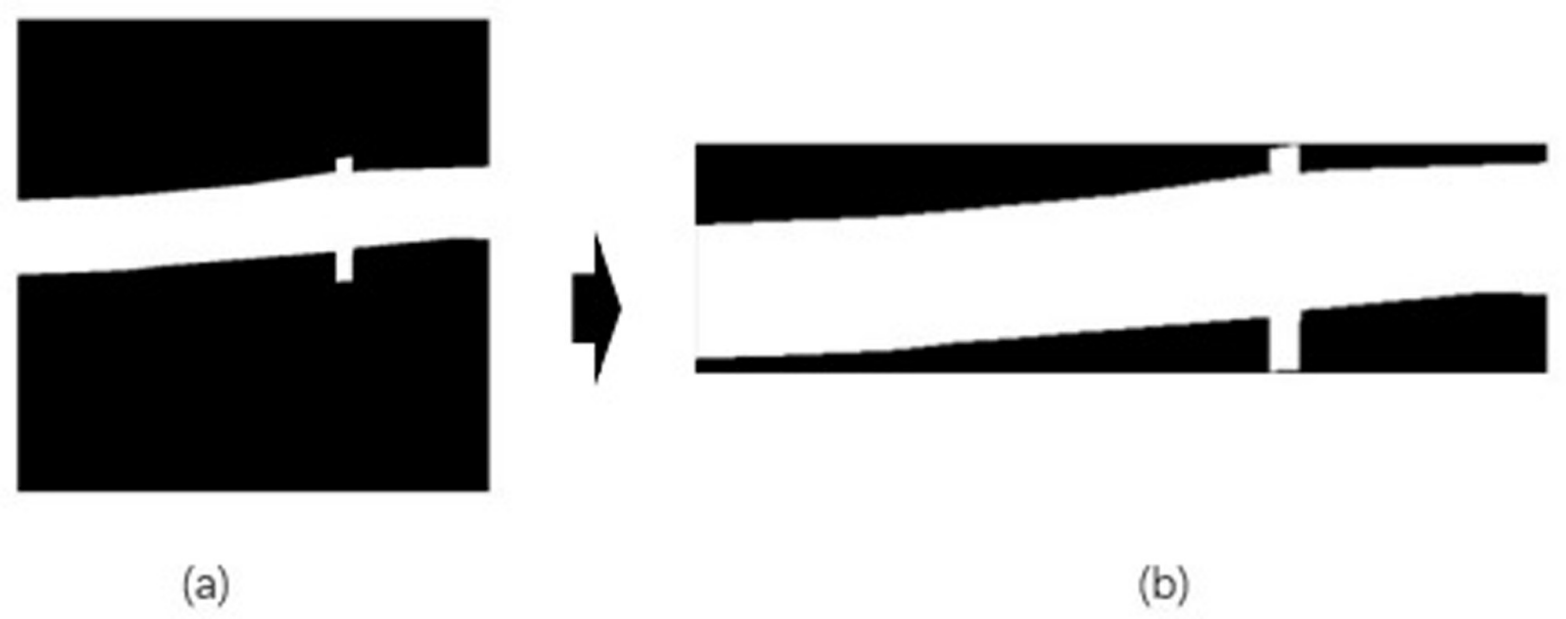
Image comparison before and after correction.

The corrected binarized image obtained can be seen as follows (Fig 14).

Upon receiving the corrected image, it can be seen that the horizontal and vertical directions of the image are very obvious. To make the image more intuitive, it is quantized, mainly by random projection in the direction perpendicular to the Hough line, in order to get the pixel frequency filtering of the ROI region. Based on the probabilistic Hough line transformation function, the contour lines of the ROI region are obtained with the following parameters: MinLineLength = 5, MaxLineGap = 10.

**Fig 15:**
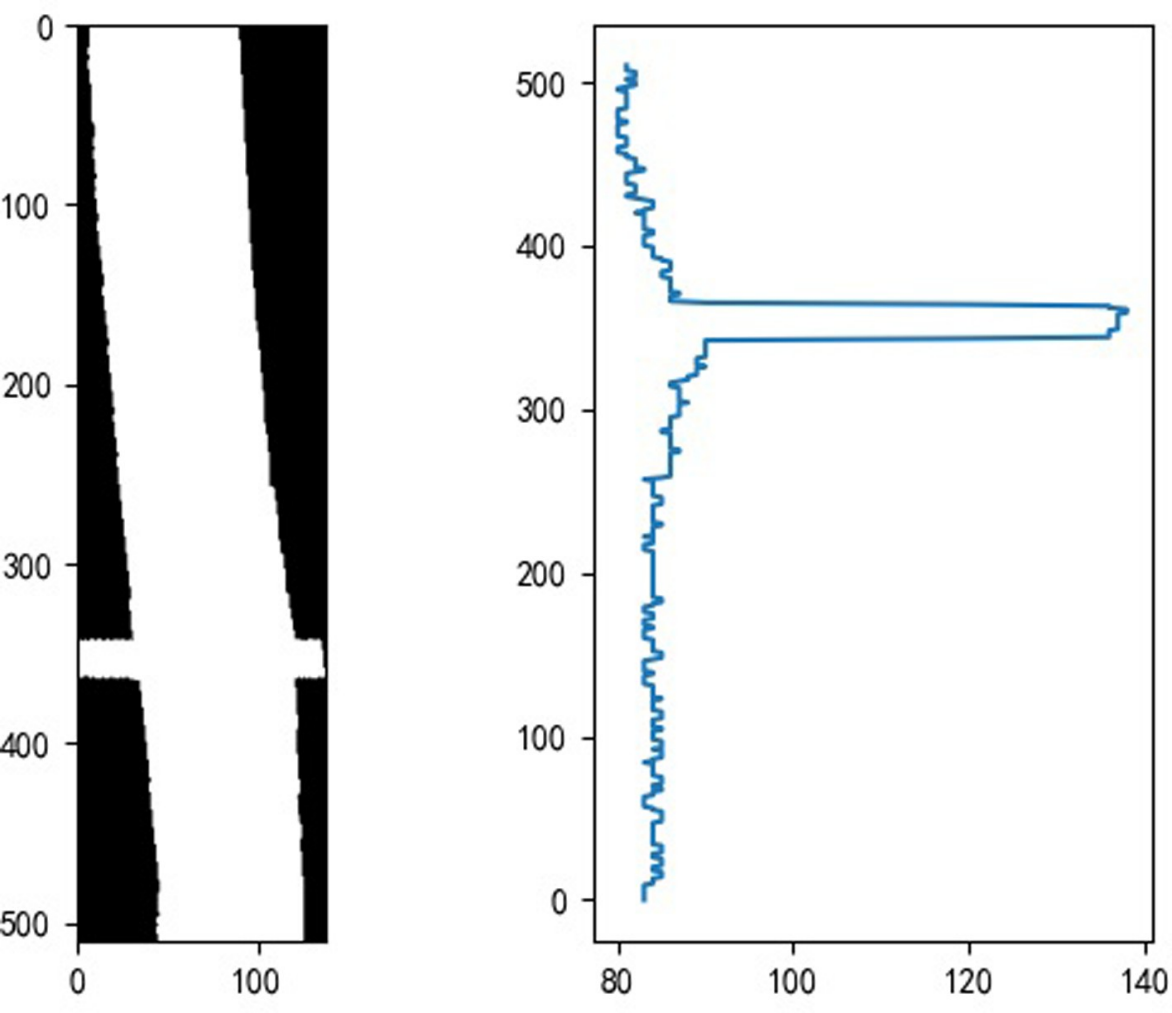
Frequency curve obtained by projection.

Through the use of Random transform, the undulating transitions caused by noise can be eliminated, while the projection of the image in the space can achieve the projection of any angle of the target image. Based on the characteristics of the boundary contour of the ROI region, the random projection transform is performed perpendicular to the Hough line. The figure illustrates the results of the Hough line detection and frequency filtering obtained by the transform (Fig 15).

#### Wavelet transform-based anomaly detection

**Fig 16:**
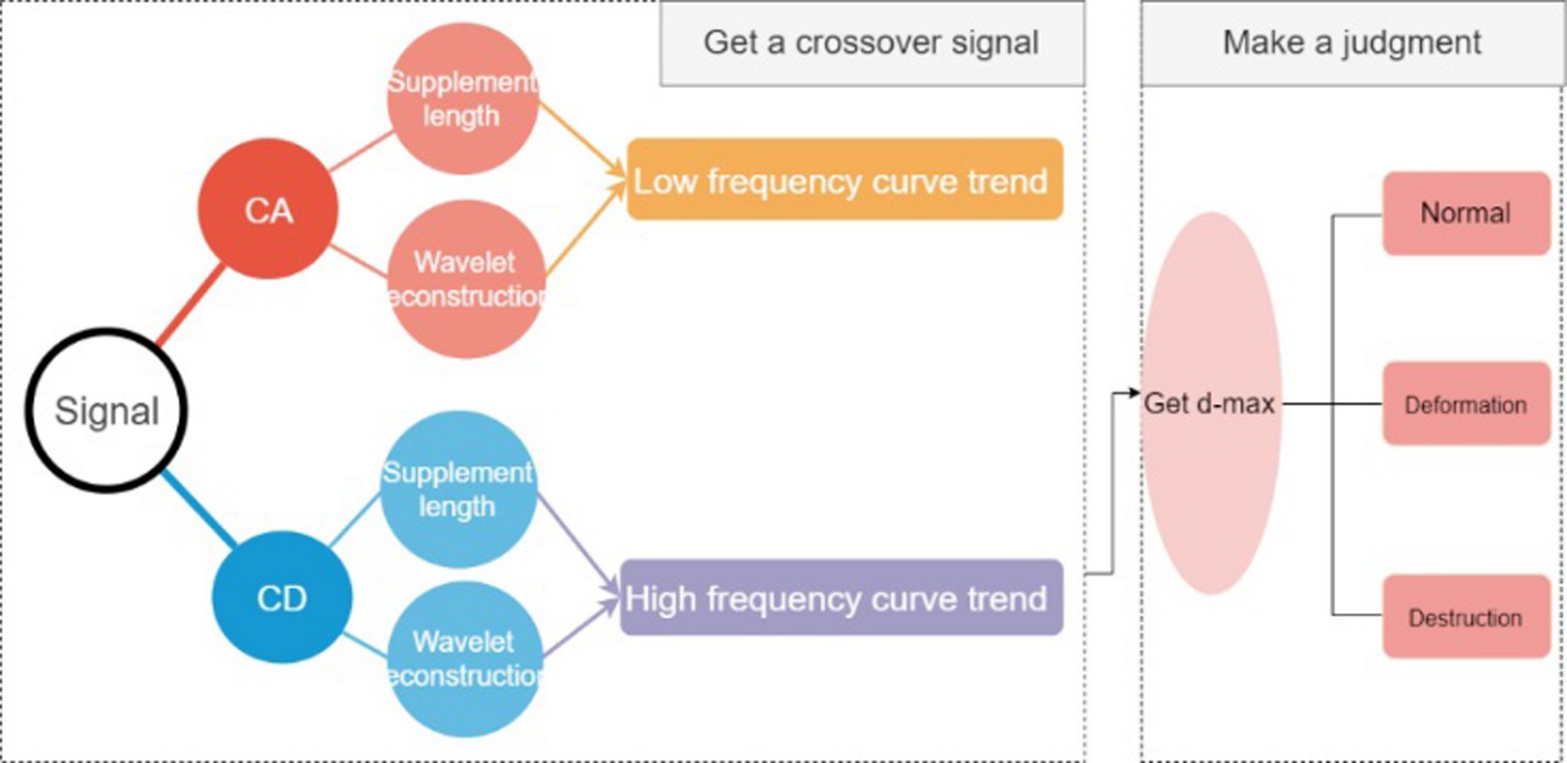
Steps for detecting anomalies.

With the wavelet transform, the original signal can be decomposed into different frequency bands with different resolution levels to obtain smooth and detail signals, and each detail signal corresponding to a different frequency band corresponds to a different detail coefficient and approximation coefficient [31]. A wavelet transform is used to decompose a time-domain signal into the high-frequency frequency band in the first order [32–35]. For marking and reference purposes, the detail coefficients of the high-frequency frequency band are analyzed, their plurality is sought, and the guardrails of different states are summarized based on the magnitude of these detail coefficients, and the main process of this method is described in figure (Fig 16).

In the frequency domain of a target image, the variation pattern in space is reflected in large part [36–38], and this is converted to a time domain signal by converting its length in space to the sampling frequency, and decomposing the original function into low-pass and high-pass functions by an analytical filter set [39–42]. Both of these functions can be expressed as

$$\left\{\begin{array}{l} d_{j}(n)=\sum_{n=1}^{N}h_{\psi }(n-2m)c_{j+1}(n)\\ c_{j}(n)=\sum_{n=1}^{N}h_{\varphi }(n-2m)c_{j+1}(n) \end{array}\right.$$ (11)

**Fig 17:**
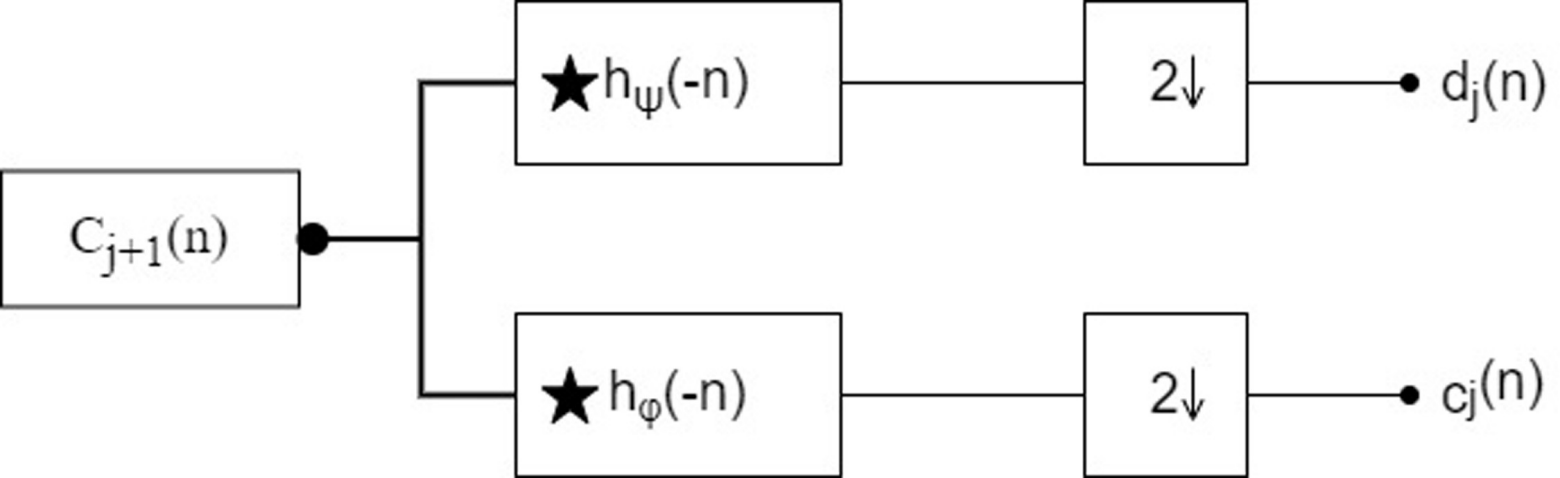
Analytical filter bank.

In this calculation, *h_ψ_*, *h_φ_* represents the high-pass and low-pass filters, n represents the number of current sampling points, j represents the number of layers in the wavelet decomposition, and m represents the degree of discretization of the wavelet function (Fig 17).

We perform a primary filtering procedure to obtain the first-order approximation coefficients and detail coefficients of the initial signal, which are supplemented with lengths for wavelet reconstruction.


$$S=\sum_{n=1}^{N}a_{j}(n)\varphi_{j,n}(t)+\sum_{n=1}^{N}d_{j}(n)\psi_{j,n}(t)$$ (12)

**Fig 18:**
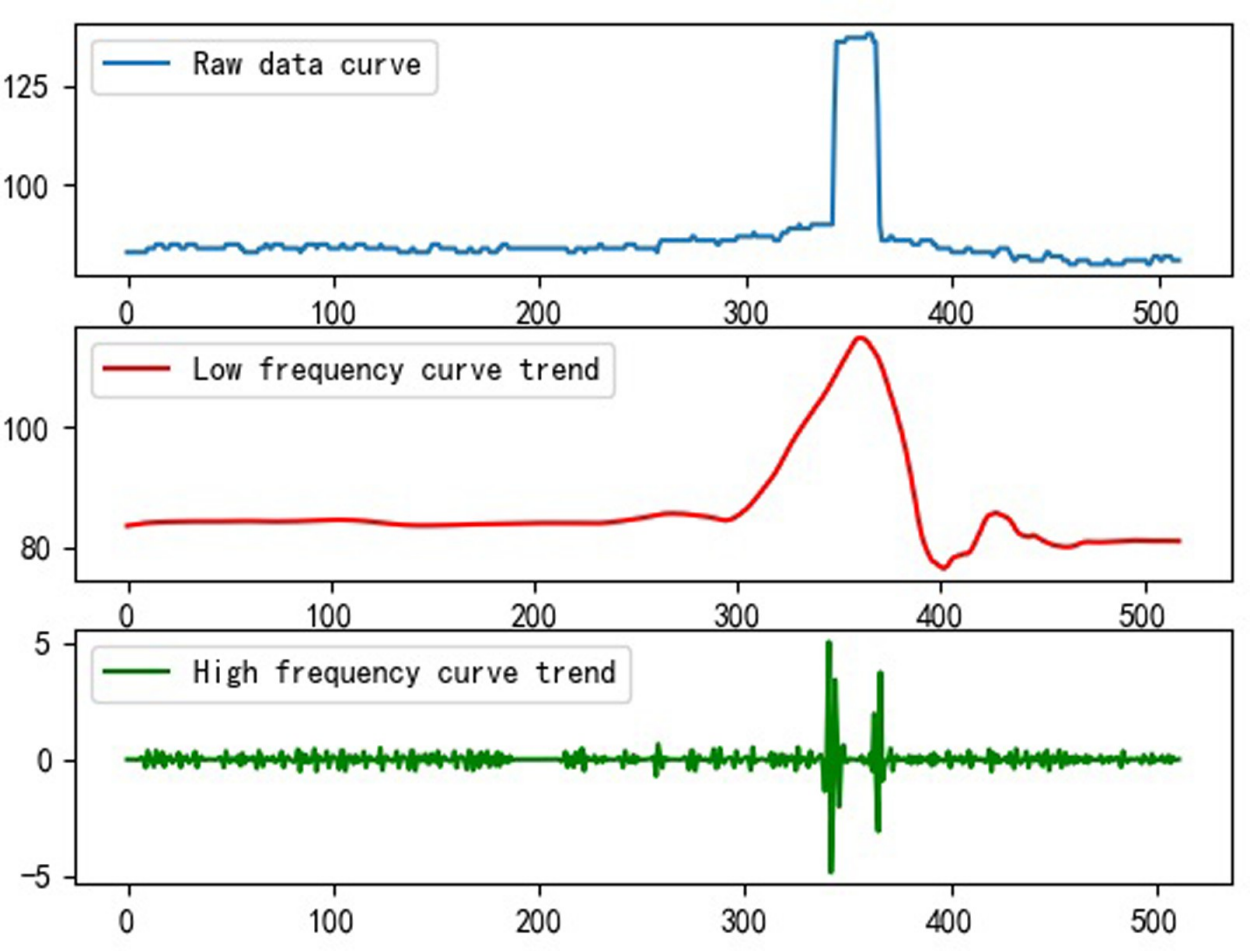
Transformation trend of diferrent signal.

In this equation, φ_*j*,*n*_(*t*) and *ψ_j,n_*(*t*) represent the scale function and wavelet function, n is the number of sampling points, and j is the number of decomposition layers, where j = 1. The signal after reconstruction fully reflects the time-frequency characteristics of the original signal as well as the magnitude of each frequency band component, as well as the trend of the signal in the high frequency band and the trend of the signal in the low frequency band of the original signal (Fig 18).

The high frequency signal in the image is more reflective of the trend of edge changes, as well as details and noise, whereas the low frequency signal is more reflective of the internal content of the edge [43–45], which reflects the overall outline and contour of the image. Consequently, the main examination of the change in the detail coefficient d is based on the deformation and damage of the highway waveform beam guardrail, as reflected in the contour characteristics. As a result, the maximum value of the detail coefficient d for different types of guardrail defects will be compared, due to the availability of more data, and will be presented in Table 5 below:

**Table 5:** Comparison of maximum detail coefficient under different defects.

Type of disease	d-max						
Normal	48.78	60.7	75	45	72.72	66.55	56.14
Deformation	18	23.79	25.27	23.21	23.18	22.56	30.45
Destruction	3.17	8.21	9.2	12.64	5.69	6.25	10.78

**Fig 19:**
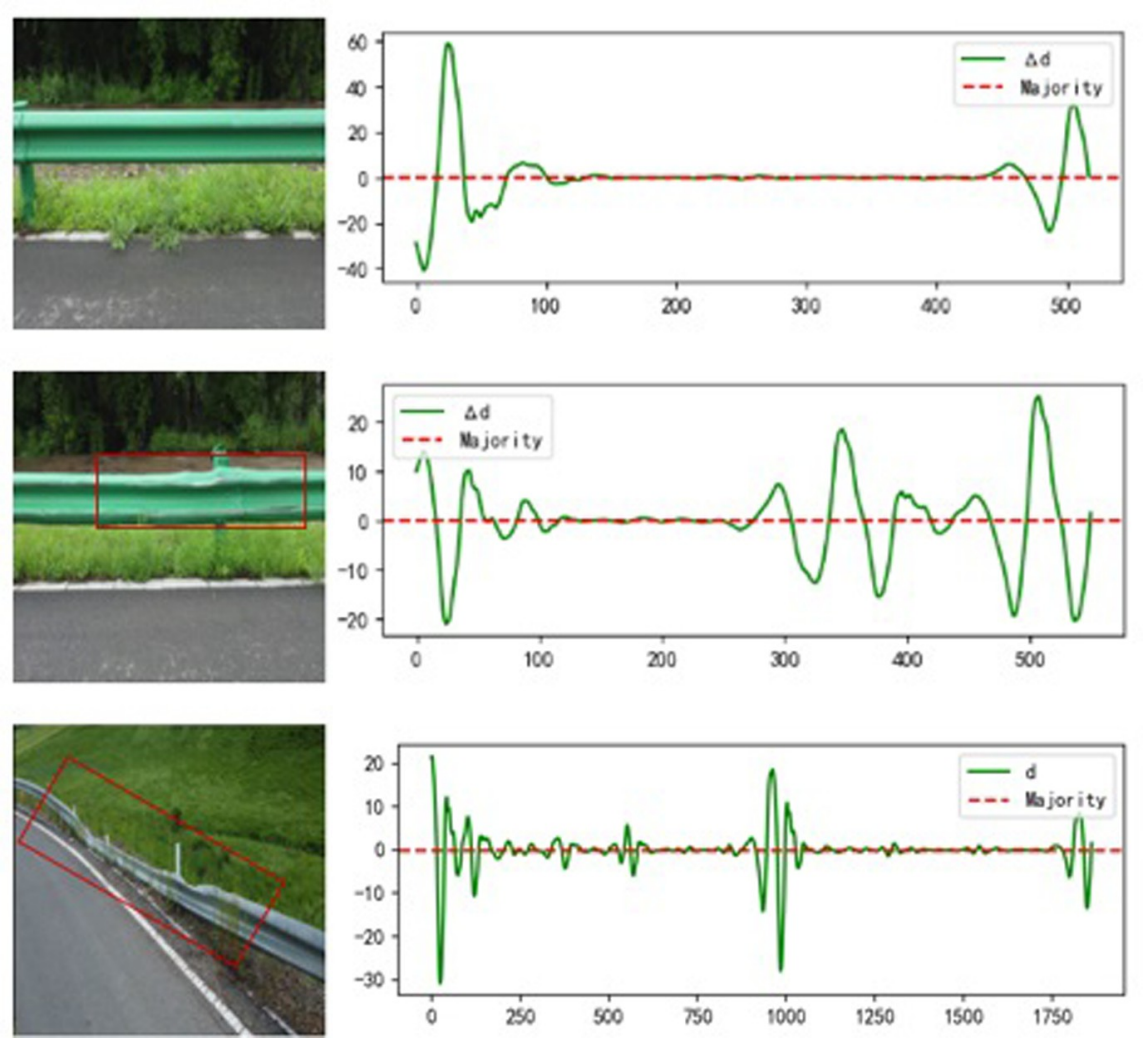
Comparison of normal, deformed and damaged wavelengths.

The data in the table indicate that the maximum detail coefficients for different types of defects are located at varying intervals; therefore, based on the size of the coefficient, the law of wavelength, and three types of guardrails, the image results obtained (Fig 19).

It shows three kinds of guardrails, each corresponding to a wave band, where the wave band illustrates a change in edge details. Normal guardrails exhibit a more regular wave, with a greater amount of undulation at the transition point between waveform plate and column, resulting in a higher edge coefficient. Deformation of the guardrail wave is more undulating, and the deformation at different positions influences the edge coefficient. In the area of deformation, more fluctuations will occur, and the detail coefficient will be smaller than normal guardrail changes. This characteristic is more apparent with damaged guardrails in terms of performance. As the guardrail is destroyed, the edge irregularity shows up in the wavelength for the wavelength the change is frequent, which leads to a small coefficient with frequent changes.

The wavelength of the guardrail has different characteristics, and each state corresponds to a different size of the detail coefficient. The wavelength and maximum detail factor of the guardrail are corresponded and summarized to determine the state of the guardrail at different intervals of the d-max value, as shown in Table 6.

**Table 6:** The d-max range of different types of defects.

Type of disease	Normal	Deformation or Destruction
Range	35–90	[0–35]∪[90–∞]

#### Defect detection experimental results

Furthermore, 1000 images of other pictures were selected for inclusion in the experiment, of which 440 were free of defects, and three types of defects were assessed in terms of corrosion layer peeling, deformation, and damage, and the corresponding indicators are shown in the Table 7 below.

**Table 7:** Experimental results of defect detection.

Type	Success	Failure	Accurary(%)	Precision	Recall
Normal	410	30	93.18	92.68%	97.43%
Abscission	232	20	92.06	86.2%	95.2%
Deformation	164	18	90.1	91.4%	94.9%
Destruction	110	16	87.3	81.8%	93.75%

According to the results, the accuracy of detection of corrosion layer peeling, deformation, and damage is 92.06%, 90.1%and 87.3%, respectively. According to the results of the experiment, the method of the study is effective at detecting guardrail defects.

**Fig 20:**
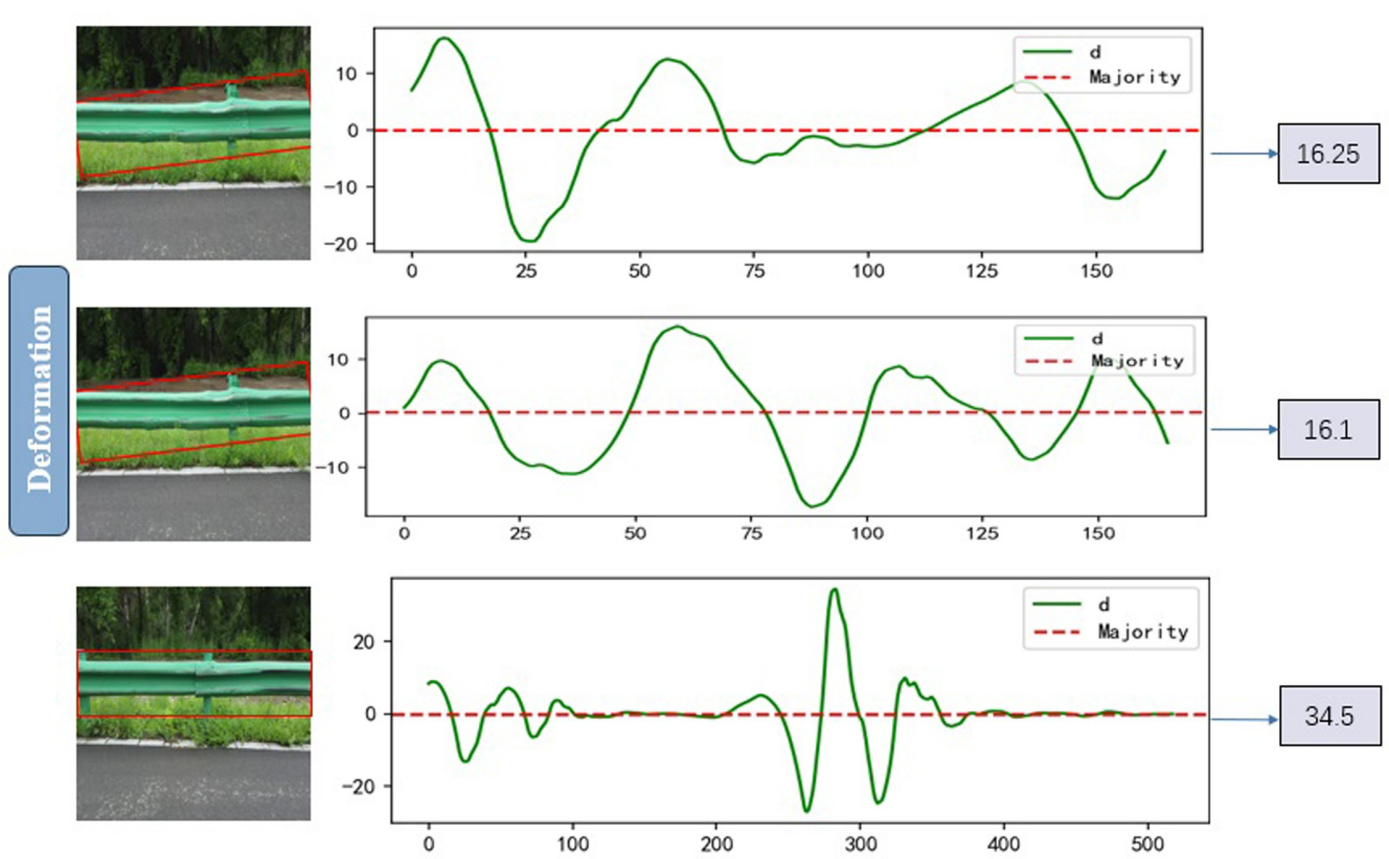
Detail coefficients for various diseases.

In addition, the results of different types of sample data are displayed (Fig 20).

From the above experimental results, it can be seen that the change of the detail coefficient curve of the normal guardrail has a certain regularity, and the detail coefficient is also normal in the flat guardrail part. And there is a deformed guardrail, the wavelength change of detail fluctuations are more obvious and the changes are more frequent and irregular, so the detail coefficient is mainly concentrated in the position of large data and small data, if there is a damaged guardrail, the detail change is very large, so there will be a high detail coefficient.

## Conclusion and future works

Using U-net improved network model for highway waveform beam guardrail segmentation, and compared with traditional and commonly used methods. The results segmented by the improved model can also be used to identify two types of defects in highway guardrails, which contributes to a new strength in highway maintenance and can greatly improve the efficiency and accuracy of highway inspections and maintenance. The paper also presents its own highway guardrail dataset, acquires images of both the near and far sides of the road, and performs enhancements and optimizations of the images, which are more in line with the actual road conditions. The main results of this study are:

1. Instead of the ordinary convolutional network of bottlenecks, the stepped HDC model can increase the perceptual field and capture contextual information at multiple scales while improving close-up segmentation. In the hybrid loss function, the model is trained on different scales of image data in the dataset and the problem of the model affecting segmentation accuracy is reduced due to the unbalanced dataset. Thus, compared with the traditional model structure, the improved model improved 8.63% and 17.67% in Miou and Dice, respectively, and decreased 2.01 in HD, which suggests that the segmentation of the improved model has greater accuracy and the segmented edges are more accurate.
2. When it comes to the problem of the anticorrosive layer peeling off highway guardrail, the partial mean value in the gray histogram of the target image is used to determine whether or not the guardrail image has this defect.
3. As part of the treatment of guardrail deformation and damage, image information is converted into projection information by random projection along the Hough line vertically, and high frequency and low frequency signal trends are determined using a wavelet transform. A maximum value of the first-order detail coefficient is calculated for each state of guardrail, which differs in terms of characteristics and detail coefficients. Therefore, the guardrail of different states and their corresponding maximum detail coefficient correspond, and the guardrail of different states is determined by the maximum coefficient.

Additionally, the guardrail images outside some data sets were selected to detect the defective disease, and the results demonstrated that the proposed detection method was able to detect the defects with greater than 85% accuracy, demonstrating its effectiveness.

As part of future research, the diversity of the data set in segmented guardrails should be further enhanced, and problems of shadowing and occlusion encountered during data collection should be addressed to improve the model’s accuracy; In the detection of anticorrosion layer shedding, for the shedding of a large area of the image there will be incomplete segmentation, thus affecting the subsequent judgement; in the detection of deformation, the shooting angle and level of the sample image is a certain requirement. As a result, further research and practice should be carried out to solve the above problems, refine the degree and level of defects, and provide more detailed advice for later maintenance.

## Supporting information

S1 Data (ZIP)
